# Draft Genome Sequence of *Pseudomonas pelagia* CL-AP6, a Psychrotolerant Bacterium Isolated from Culture of Antarctic Green Alga *Pyramimonas gelidicola*

**DOI:** 10.1128/genomeA.00699-13

**Published:** 2013-09-05

**Authors:** Hye Yeon Koh, Woongsic Jung, Hackwon Do, Sung Gu Lee, Jun Hyuck Lee, Hak Jun Kim

**Affiliations:** Division of Polar Life Sciences, Korea Polar Research Institute, Incheon, Republic of Koreaa; Department of Polar Sciences, University of Science and Technology, Incheon, Republic of Koreab; Department of Applied Marine Biotechnology and Engineering, Gangneung-Wonju National University, Gangneung, Republic of Koreac; Department of Chemistry, Pukyong National University, Busan, Republic of Koread

## Abstract

*Pseudomonas pelagia* CL-AP6, isolated from a culture of the Antarctic green alga *Pyramimonas gelidicola*, is a psychrotolerant bacterium. Here, we report the draft genome sequence of this strain, which may provide insights into the mutualistic interaction between microalgae and bacteria in sea ice, as well as the cold adaptation mechanisms of bacteria.

## GENOME ANNOUNCEMENT

*Pseudomonas pelagia* CL-AP6 was isolated from a culture of the Antarctic green alga *Pyramimonas gelidicola* that was established in 1981 and maintained at 2 to 4°C under continuous light conditions for more than three decades (1). This bacterium is Gram-negative, strictly aerobic, rod-shaped, and motile by means of a single polar flagellum. Like other microalgae and bacteria cocolonized in sea ice, *P. pelagia* CL-AP6 may be trapped in sea ice with *P. gelidicola* (2, 3). To cope with cold stresses, *P. gelidicola* seemed to upregulate the expression of antifreeze proteins, which are essential for survival in ice (4). However, *P. pelagia* exhibited no antifreeze activity (W. Jung and H. J. Kim, unpublished data), implying that this strain may take advantage of the secreted antifreeze proteins from coexisting *P. gelidicola*. The genome sequence of the bacterium may provide insights into the mutualistic interaction between microalgae and bacteria in sea ice.

The genome of *P. pelagia* CL-AP6 was analyzed using the Illumina HiSeq 2000 (San Diego, CA) with a 300-bp paired-end library (6,884,732 reads). The Illumina sequencing achieved about 223-fold coverage. The reads generated by Illumina HiSeq 2000 were assembled using the CLC Genomics Workbench v5.0 (CLC bio), and the resulting contigs were curated by CodonCode Aligner v3.7 (CodonCode Co.). Gene prediction and annotation were performed using Glimmer3, tRNAscan, EzTaxon-e with hidden Markov model searching, NCBI reference sequences, and several open databases (5–11). The draft genome of *P. pelagia* CL-AP6 (about 4,642,307 bp) contains 81 contigs, with an N_50_ contig length of approximately 186,161 kb. The G+C content is 57.41%. A total of 4,314 protein-encoding genes and 46 tRNA genes were annotated in the draft genome. Three rRNA operons were predicted based on the sequence coverage value. Approximately 89% of nucleotides were predicted to be protein-coding regions, and 2,640 (60.5%) protein-coding sequences were annotated with known proteins. A comparison of genome sequences (12) showed that *Pseudomonas aeruginosa* strain UCBPP-PA14 (genome ID 208963.12, score 507) and *P. aeruginosa* PAO1 (genome ID 208964.12, score 489) are the closest neighbors of *P. pelagia* CL-AP6. We found genes for cold shock DEAD box protein A and cold shock protein Csp C that are involved in the cold adaptation mechanism. The genome sequence of *P. pelagia* CL-AP6 may be useful for exploring the mutualistic interaction between microalgae and bacteria in sea ice, as well as cold adaptation mechanisms of bacteria.

### Nucleotide sequence accession numbers.

This whole-genome shotgun project has been deposited at DDBJ/EMBL/GenBank under the accession no. AROI00000000. The version described in this paper is the first version, AROI01000000.
